# Can Arbuscular Mycorrhizal Fungi Reduce the Growth of Agricultural Weeds?

**DOI:** 10.1371/journal.pone.0027825

**Published:** 2011-12-02

**Authors:** Rita S. L. Veiga, Jan Jansa, Emmanuel Frossard, Marcel G. A. van der Heijden

**Affiliations:** 1 Ecological Farming Systems, Agroscope Reckenholz-Tänikon Research Station ART, Zürich, Switzerland; 2 Plant-Microbe Interactions, Institute of Environmental Biology, Faculty of Science, Utrecht University, Utrecht, The Netherlands; 3 Institute of Agricultural Sciences, Swiss Federal Institute of Technology (ETH) Zürich, Lindau, Switzerland; University of Hull, United Kingdom

## Abstract

**Background:**

Arbuscular mycorrhizal fungi (AMF) are known for their beneficial effects on plants. However, there is increasing evidence that some ruderal plants, including several agricultural weeds, respond negatively to AMF colonization. Here, we investigated the effect of AMF on the growth of individual weed species and on weed-crop interactions.

**Methodology/Principal Findings:**

First, under controlled glasshouse conditions, we screened growth responses of nine weed species and three crops to a widespread AMF, *Glomus intraradices*. None of the weeds screened showed a significant positive mycorrhizal growth response and four weed species were significantly reduced by the AMF (growth responses between −22 and −35%). In a subsequent experiment, we selected three of the negatively responding weed species – *Echinochloa crus-galli*, *Setaria viridis* and *Solanum nigrum* – and analyzed their responses to a combination of three AMF (*Glomus intraradices*, *Glomus mosseae* and *Glomus claroideum*). Finally, we tested whether the presence of a crop (maize) enhanced the suppressive effect of AMF on weeds. We found that the growth of the three selected weed species was also reduced by a combination of AMF and that the presence of maize amplified the negative effect of AMF on the growth of *E. crus-galli*.

**Conclusions/Significance:**

Our results show that AMF can negatively influence the growth of some weed species indicating that AMF have the potential to act as determinants of weed community structure. Furthermore, mycorrhizal weed growth reductions can be amplified in the presence of a crop. Previous studies have shown that AMF provide a number of beneficial ecosystem services. Taken together with our current results, the maintenance and promotion of AMF activity may thereby contribute to sustainable management of agroecosystems. However, in order to further the practical and ecological relevance of our findings, additional experiments should be performed under field conditions.

## Introduction

Weeds represent one of the most serious problems in crop production, with a potential crop loss of up to 34% each year [1]. In conventional farming systems, actual losses to weeds have been kept to considerably lower values mainly through intensive tillage and herbicide application [2]. However, with the increasing restrictions on chemicals use (e.g. European Union pesticide review EU 91/414/EEC), the emergence of more ecologically sound farming systems (e.g. Organic Farming) and the recognition of the importance of weeds to maintain or enhance on-farm biodiversity [3], focus has shifted to sustainable alternative approaches to weed management. The use and manipulation of organisms that selectively cause damage to weeds has long been recognized as one of such alternatives [4], [5] but not much attention has been paid to soil biota despite its known influence on weed biology and ecology [6]. Here, we focus on weed interactions with a particular group of symbiotic soil fungi, the arbuscular mycorrhizal fungi (AMF).

Arbuscular mycorrhizal (AM) symbiosis is an ancient association formed between fungi from the phylum Glomeromycota and almost two thirds of all land plants [7], [8]. The AMF extraradical mycelium acts as an extension of the host root system, taking up nutrients (especially phosphate) which are delivered to the plant in r**e**turn for photosynthetically assimilated carbon [8], [9]. Thus, it is to be expected that a host plant will benefit directly from the AM symbiosis through increased nutrient uptake, and, consequentially, increased growth. However, this is not always the case; some plants do not show any growth increase while others even seem to be negatively affected by AMF colonization [10]–[12].

In agroecosystems, the effects of AMF on crops have been thoroughly studied [8], [13]–[16]. These studies show that AMF mainly promote crop yield under nutrient deficient conditions, although negative or no effects have also been reported [17]–[21]. Less is known about the interactions between AMF and agricultural weeds. It has been suggested that negative effects of AMF are more likely to occur in ruderal species colonizing early successional environments where there is considerable disturbance and where AMF are sometimes absent [22]–[24]. Many weeds have a ruderal lifestyle and colonize agroecosystems, which are often heavily disturbed environments where AMF abundance and diversity has been shown to be reduced by practices like monocropping, tillage and fertilization [25]–[27]. Therefore, it is plausible to suggest that AMF-weed interactions might not be of the mutualistic type. Vatovec et al. [28] and Jordan & Huerd [29] found variable weed responses to soil fungi, including negative responses. More recently, Rinaudo et al. [30] showed that weeds grown in community were suppressed by AMF and that this effect was even stronger in the presence of a crop plant (sunflower). However, it is still unclear how individual weed species respond to AMF and how individual responses are affected by the presence of a crop. The potential role of AMF in weed management has already been discussed [31], [32] but, in order to realize this potential, a better understanding of the effects of AMF on individual weed species is required.

In this study we analyzed individual mycorrhizal growth responses of nine weeds (listed in Table 1) that are common in many countries, including Switzerland [33], and recognized by farmers as troublesome in wheat and/or maize cultures by reducing yield and quality of grain (based on interviews with farmer advisors at Agroscope Reckenholz-Tänikon Research Station ART). We first tested whether the widespread AMF species *G. intraradices* [34] reduces the growth of the selected weeds. Under field conditions, though, usually more than one AMF species coexist in the host roots [34]–[36] and plants can respond differently to different AMF or to different levels of AMF diversity [12], [37], [38]. For these reasons, we then tested whether the negative effects of *G. intraradices* on weed growth are reproducible when the weeds are co-inoculated with other AMF species. Finally, we tested whether the negative effects of AMF on weeds are enhanced by the presence of a crop plant.

**Table 1:** Plant species used in experiments 1 and 2 with the respective common name and family.

Exp	Crop species	Common name	Family
1	*Trifolium pratense* L.	red clover	Fabaceae
1	*Triticum aestivum* L.	common wheat	Poaceae
1, 2	*Zea mays* L.	maize, corn	Poaceae

	Weed species		
1	*Agropyron repens* (L.) P. Beauv.	quackgrass	Poaceae
1	*Alopecurus myosuroides* Huds.	slender meadow foxtail, blackgrass	Poaceae
1	*Apera spica-venti* (L.) P. Beauv.	loose silkybent	Poaceae
1	*Cirsium arvense* (L.) Scop.	canada thistle, creeping thistle	Asteraceae
1	*Digitaria sanguinalis* (L.) Scop.	hairy crabgrass, large crabgrass	Poaceae
1, 2	*Echinochloa crus-galli* (L.) P. Beauv.	barnyardgrass	Poaceae
1	*Poa annua* L.	annual bluegrass	Poaceae
1, 2	*Setaria viridis* (L.) P. Beauv.	green bristlegrass, green foxtail	Poaceae
1, 2	*Solanum nigrum* L.	black nightshade	Solanaceae

## Materials and Methods

### Ethics Statement

No specific permits were required for the described studies. The soil used was collected from a field at Agroscope Reckenholz-Tänikon Research Station ART and the experiments were run in glasshouses also at Agroscope Reckenholz-Tänikon Research Station ART. Agroscope Reckenholz-Tänikon Research Station ART belongs to the Swiss Federal Office for Agriculture and is not privately owned or comprises protected area. The experiments did not involve endangered or protected species.

### Methods

In this paper we present two experiments. In the first experiment we analyzed the individual growth responses of nine agricultural weed species (Table 1) to the presence of the AMF *G. intraradices*. We added three crop species - wheat, maize and red clover – to compare their mycorrhizal growth responses to the ones of the weeds. Clover, in particular, as most of the legumes, often shows a positive response to AMF [11], [39], [40], hence constituting a positive control. In a second experiment we tested the effect of a combination of AMF species on weed growth responses and investigated AMF-weed-crop interactions: out of the nine weeds, we selected three species showing a negative growth response in the first experiment (*Echinochloa crus-galli*, *Setaria viridis* and *Solanum nigrum*) to analyze their responses in the presence of maize and to a combination of AMF species (*G. intraradices*, *G. claroideum* and *G. mosseae*). These three *Glomus* sp. are common and often co-occurring in Swiss arable soils [25], [36]. Since direct root competition for soil resources might hinder interpretation of results, we physically separated roots of maize from weed roots by dividing the pots with a 30 µm mesh which still allows the AM hyphae to pass through.

Both experiments were performed under controlled conditions in the glasshouse and thereby might differ from a field situation. Nonetheless, we consider such experiments as an important starting point for further research on the effects of AMF on weeds in the field.

### Plant material, fungal inoculum and soil substrate

Plant species used in both experiments are listed in Table 1. Wheat (*Triticum aestivum* L. cv. Runal), maize (*Zea mays* L. cv. Gavott) and red clover (*Trifolium pratense* L. cv. Milvus) seeds were obtained from Agroscope Reckenholz-Tänikon Research Station ART, Switzerland. Seeds of the weed species were obtained from Herbiseed, UK (www.herbiseed.com). All seeds were surface sterilized in 1.25% sodium hypochlorite for 10 min and subsequently rinsed with dH_2_O.

In experiment 1, soil inoculum containing colonized roots and spores of *Glomus intraradices* Schenck & Smith (BEG 21) [41] was used. In experiment 2, a mixture of equal parts in weight of *G. intraradices* (BEG 21), *Glomus claroideum* Schenck & Smith (HG 181) and *Glomus mosseae* (Nicol. & Gerd.) Gerd. & Trappe (HG 505) soil inocula constituted the AMF inoculum that was added to the pots. The *G. claroideum* and *G. mosseae* isolates were kindly provided by Hannes Gamper (University of Basel). These isolates originated from single spores collected in 2002 from trap cultures in grassland plots. Grassland plots were established in previously arable land in the Swiss long-term Free-Air CO_2_ Enrichment (FACE) experiment in Eschikon, Switzerland (see [42], [43] for more details of the site). All inocula were propagated as pure cultures on *Plantago lanceolata* L. for 5 months, in pots filled with an autoclaved (99 min at 121°C) mixture of quartz sand with 20% (v∶v) field soil. *Glomus intraradices*, *G. claroideum* and *G. mosseae* colonized 77%, 43% and 51% of the root length of *P. lanceolata*, respectively, as assessed microscopically after staining with trypan blue (see details of the method below). The non-mycorrhizal (NM) control inoculum consisted of the same soil inocula as mentioned above, for experiments 1 and 2, but sterilized by autoclaving (2× 99 min at 121°C).

The soil substrate used for both experiments consisted of an autoclaved (99 min at 121°C) mixture of 50% (v∶v) field soil with quartz sand. Field soil was collected from an organically managed field plot at Agroscope Reckenholz-Tänikon Research Station ART (Zurich, Switzerland) but at different time points for experiments 1 and 2. The pH and primary plant-available nutrient concentrations of the soil substrate for each experiment are shown in the Supporting Information (Table S1). Note that both soil substrates were P-rich.

### Experiment 1: mycorrhizal growth responses of nine weed species (screening)

This experiment was set up in a randomized factorial design with nine weed and three crop plant species inoculated with *G. intraradices* (AMF treatment) or with NM control inoculum. Each treatment was replicated six times for a total of 144 pots (experimental units). The position of the pots in the glasshouse was randomized every 2 weeks.

Pots were filled with 0.6 L of autoclaved soil substrate with 7% (v∶v) *G. intraradices* soil inoculum or the same amount of sterilized inoculum in NM control pots. All the pots received 5 mL of inoculum washing (120 g of soil inoculum suspended in 1 L water and filtered through Whatman filter paper) to correct for possible differences in microbial communities.

Seeds were germinated in quartz sand for approximately 4–7 days (depending on the plant species). Germinated seeds were transferred into pots and thinned afterwards, leaving two seedlings per pot (except for maize and wheat where only one seedling was left per pot).

Pots were watered three times a week with dH_2_O, and every pot was adjusted weekly to 10% water content by weighing. Plants were maintained in the glasshouse with constant temperature (25°C) and constant lighting provided by 400 W high-pressure sodium lights to a daylength of 14 h. Plants were harvested 8 weeks after planting.

### Experiment 2: AMF-Weed-Crop interactions

This experiment was set up in a randomized factorial design with three weed (*E. crus-galli*, *S. viridis* and *S. nigrum*) and one crop species (maize) grown either alone (named “monocultures” hereafter), or each of the weed species grown in combination with maize (named “mixtures” hereafter). Plants were inoculated with a mixture of *G. intraradices*, *G. mosseae* and *G. claroideum* (AMF treatment) or with NM control inoculum. Each treatment was replicated seven times for a total of 98 pots (experimental units). The position of the pots in the glasshouse was randomized every 2 weeks.

Pots were divided in two equal parts by 30 µm nylon mesh to separate roots but still allowing the passage of AMF hyphae. Each half received 1 L of autoclaved soil mixture with 7% (v∶v) AMF soil inoculum or the same amount of sterilized inoculum in NM control pots. All the pots received 10 mL (5 mL each half) of inoculum washing (300 g of the soil inoculum suspended in 2 L water and filtered through Whatman filter paper).

For the mixtures, three seeds of maize were sown in one half of the pot, while five seeds of the same weed species were sown in the other half. For the monocultures, five weed seeds or three maize seeds were sown in one half while the other half remained unsown. Germinating seeds were thinned to one maize seedling or three weed seedlings per half-pot.

Pots were watered three times a week with dH_2_O and every pot was adjusted weekly to 13% water content by weighing. Plants were maintained in the glasshouse and additional lighting was provided by 400 W high-pressure sodium lights when natural light levels reached <250 W m^−2^, to a daylength of 14 h. Temperatures varied in the glasshouse between a minimum of 14°C and a maximum of 22°C. Plants were harvested 12 weeks after planting.

### Harvest and analysis

Aboveground plant parts were cut at the soil surface, oven dried (80°C) and weighed to determine the aboveground biomass. Soil was separated from plant roots and carefully washed. Roots were then cut into 1 cm segments, mixed and divided in two subsamples which were both weighed. One of the subsamples was oven dried (80°C) and weighed while the other was taken to determine the percentage of root length colonized by AMF. The belowground biomass of the subsample taken for root colonization determination was calculated by multiplying its fresh weight with the dry to fresh weight ratio of the oven-dried root subsample. Sum of the belowground biomass of both subsamples and aboveground biomass gave the total biomass for each plant species.

Mycorrhizal growth responses (MGR) were calculated using the following formulas [11]:
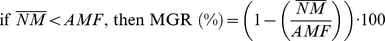


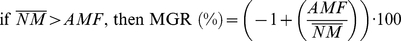
Where 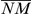 is the mean total biomass of the NM controls for each plant species, and *AMF* is the total biomass of that plant species in individual pots. A positive mycorrhizal growth response means that the plant species benefited from AMF inoculation in terms of total biomass. A negative mycorrhizal growth response indicates that the plant species was suppressed by AMF.

Root samples for measurement of AMF colonization were cleared with 10% KOH and stained with trypan blue [44]. The percentage of root length colonized by AMF and frequency of hyphae, vesicles and arbuscules was estimated according to McGonigle et al. [45], using at least 100 intersections per root sample.

### Statistical analyses

Total biomass, root length colonized by AMF (total, vesicles and arbuscules) and mycorrhizal growth responses were analyzed separately using generalized linear least squares with the gls function from the nlme library [46] for R2.9.0 [47]. Whenever there was heterogeneity in the variance structure between treatments we used the varIdent() function to allow each treatment to have a different variance. Student's t-test was used to assess differences between two sample means.

In the first experiment, root length colonized and mycorrhizal growth responses were analyzed by a one-way analysis of variance (ANOVA) with “plant species” (each of 12 plant species investigated) as factor, while total biomass was analyzed by a two-way ANOVA with “plant species” and “AMF” (AMF treatment or NM control) as factors. During this experiment, both *Agropyron repens* plants in one of the pots inoculated with NM control inoculum died and this replicate was therefore eliminated from the analysis.

In the second experiment, weeds and maize were analyzed separately. For the weeds, root length colonized and mycorrhizal growth responses were analyzed by a two-way ANOVA with “weed species” (each of the three weed species investigated) and “plant combination” (weed monocultures or mixtures with maize) as factors. Total biomass was analyzed separately for monocultures and mixtures by two-way ANOVA with “weed species” and “AMF” as factors. For the maize, root length colonized and mycorrhizal growth responses were analyzed by a one-way ANOVA with “plant combination” as a factor. As we first wanted to assess the general effect of the presence of weeds on the root length colonized and mycorrhizal growth responses of maize, we treated “plant combination” as a factor with two levels (maize monoculture and mixture with weeds). Then we treated “plant combination” as a factor with four levels (maize monoculture, maize in mixture with *E. crus-galli*, maize in mixture with *S. viridis* and maize in mixture with *S. nigrum*) to assess the differences between maize in monoculture and maize grown with each of the weed species. Maize total biomass was also analyzed separately for monoculture and mixtures by one-way ANOVA with “AMF” as factor.

Several authors use the sequential Bonferroni adjustment to correct for multiple testing [48] but there are also important flaws in this method [49]. Therefore, we present (Table S2) *P*-values for total biomass comparisons between the AMF treatment and NM control for each of the 12 plant species tested in the first experiment (where Bonferroni corrections would have affected the significance of the results), enabling readers to perform a Bonferroni adjustment if preferred. In the text and figures, we present means with their standard errors (SEM). In the Supporting Information (Tables S3, S4, S5, S6, S7, S8) we present the complete results for the ANOVA tests performed.

## Results

### Experiment 1: mycorrhizal growth responses of nine weed species (screening)

#### AMF colonization

No colonization was observed in NM control plants for any of the species. When inoculated with *G. intraradices*, the percentage of total root length colonized varied greatly among species (*F*_11,60_ = 298.7, *P*<0.0001), ranging from 2% to 97% (Table 2). All the typical fungal structures – hyphae, vesicles and arbuscules – were observed in each plant species at least once. Plant species with a low to moderate percentage of root length colonized (<50%) by *G. intraradices* included the weeds *Alopecurus myosuroides*, *Apera spica-venti*, *Poa annua* and the crop clover. The remaining six weeds and the crop species wheat and maize all showed a percentage of root colonization higher than 50%.

**Table 2:** Percentage of root length colonized by *G. intraradices* in experiment 1.

	Root length colonized (%)		
Crop species	Total	Vesicles	Arbuscules
Maize	86±2.1	44±2.0	23±3.3
Wheat	75±3.1	19±2.1	16±2.0
Clover	45±7.1	2±2.1	8±4.1

Weed species			
*Cirsium arvense*	97±2.0	32±1.6	49±4.2
*Solanum nigrum*	79±1.9	24±2.5	21±3.1
*Echinochloa crus-gali*	77±3.7	20±2.1	28±3.10
*Digitaria sanguinalis*	66±4.9	19±3.0	14±1.9
*Agropyron repens*	63±7.9	20±3.0	17±3.5
*Setaria viridis*	63±4.5	22±2.6	7±0.9
*Poa annua*	43±3.9	7±1.4	5±0.7
*Apera spica-venti*	8±4.3	1±1.0	1±0.4
*Alopecurus myosuroides*	2±1.2	0.5±0.9	0.3±0.3

Values are means of six replicates ± SEM.

#### Growth responses

**Figure 1:**
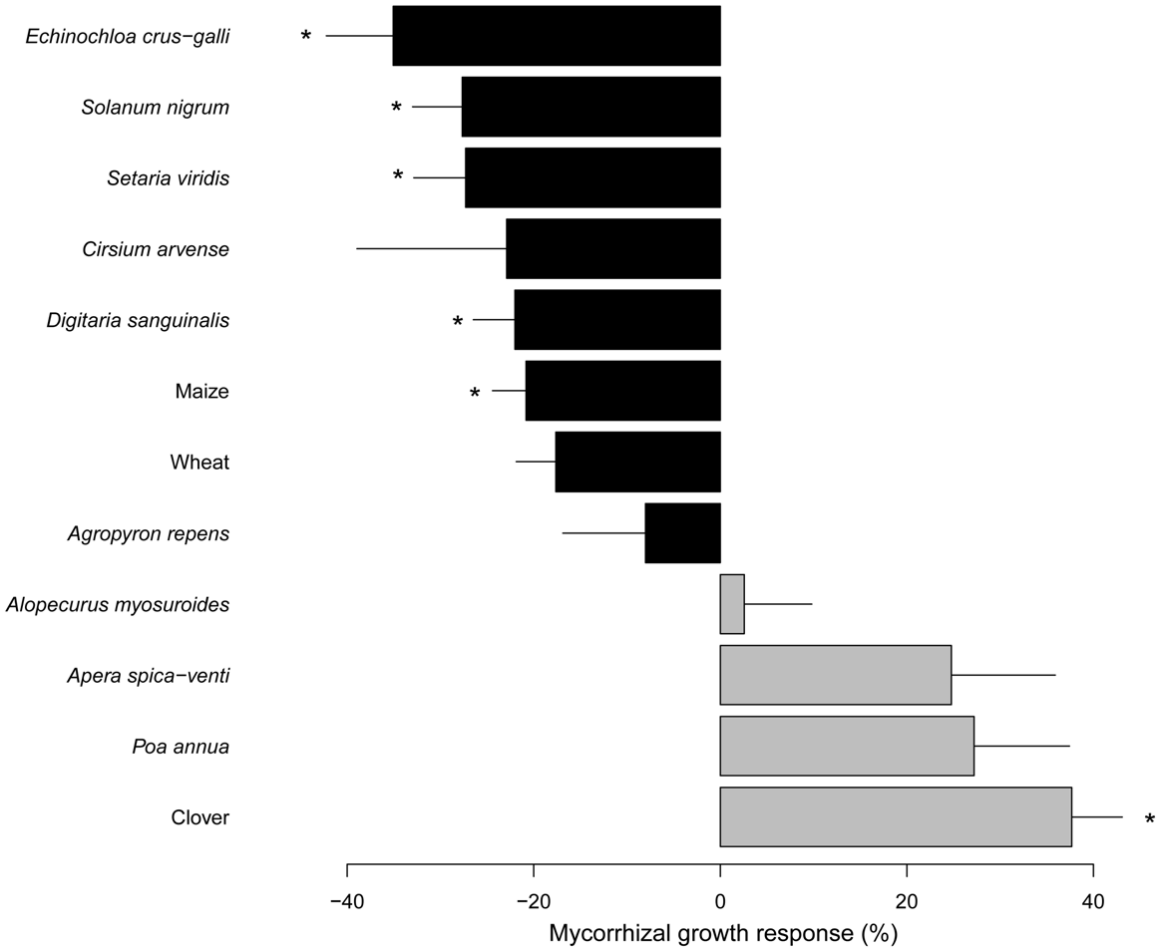
Mycorrhizal growth responses (%) of the nine weed and three crop species in experiment 1. Bars are means of six replicates ± SEM. Bars in grey indicate species with a total root length colonized by *G. intraradices* <50% while bars in black indicate species with total root length colonized by *G. intraradices* >50%. Asterisks represent significant differences (*P*<0.05) in total biomass between the AMF treatment and NM control, for each species.

The analysis of the total biomass showed that the effect of AMF was highly dependent on the plant species (significant “AMF”×“plant species” interaction *F*_11,119_ = 6.1, *P*<0.0001). This translates to variable individual mycorrhizal growth responses of the different species (*F*_11,60_ = 9.0, *P*<0.0001). Indeed, Fig. 1 shows how mycorrhizal growth responses varied among the 12 plant species from positive to negative, ranging on average from 38±5.4% in clover to −35±7.2% in the weed *E. crus-galli*. Four plants species, *A. myosuroides*, *A. spica-venti*, *P. annua* and clover, showed positive mycorrhizal growth responses. These were higher than 20% except in the case of *A. myosuroides*. The other eight plant species showed negative responses to *G. intraradices*, with six species showing a strong negative mycorrhizal growth response, lower than −20%.

Six plant species showed statistically significant differences in total biomass when inoculated with *G. intraradices*, compared to the NM controls (Table S2). The referred species were: clover (*t* = 2.5, *P* = 0.016), maize (*t* = −3.7, *P*<0.001), *Digitaria sanguinalis* (*t* = −3.5, *P*<0.001), *E. crus-galli* (*t* = −5.7, *P*<0.0001), *S. viridis* (*t* = −4.3, *P*<0.0001) and *S. nigrum* (*t* = −2.5, *P* = 0.014). From these species, clover was the only one showing a positive growth response which means that it was also the only plant whose biomass was significantly increased in the presence of the AMF *G. intraradices*. All the other five species suffered a significant biomass reduction.

### Experiment 2: AMF-Weed-Crop interactions

#### AMF colonization

No colonization was observed in NM control pots. Roots of all the four plant species (*E. crus-galli*, *S. nigrum*, *S. viridis* and maize) were colonized by AMF, showing the typical mycorrhizal structures, when inoculated with a mixture of *G. intraradices*, *G. claroideum* and *G. mosseae*.

**Figure 2:**
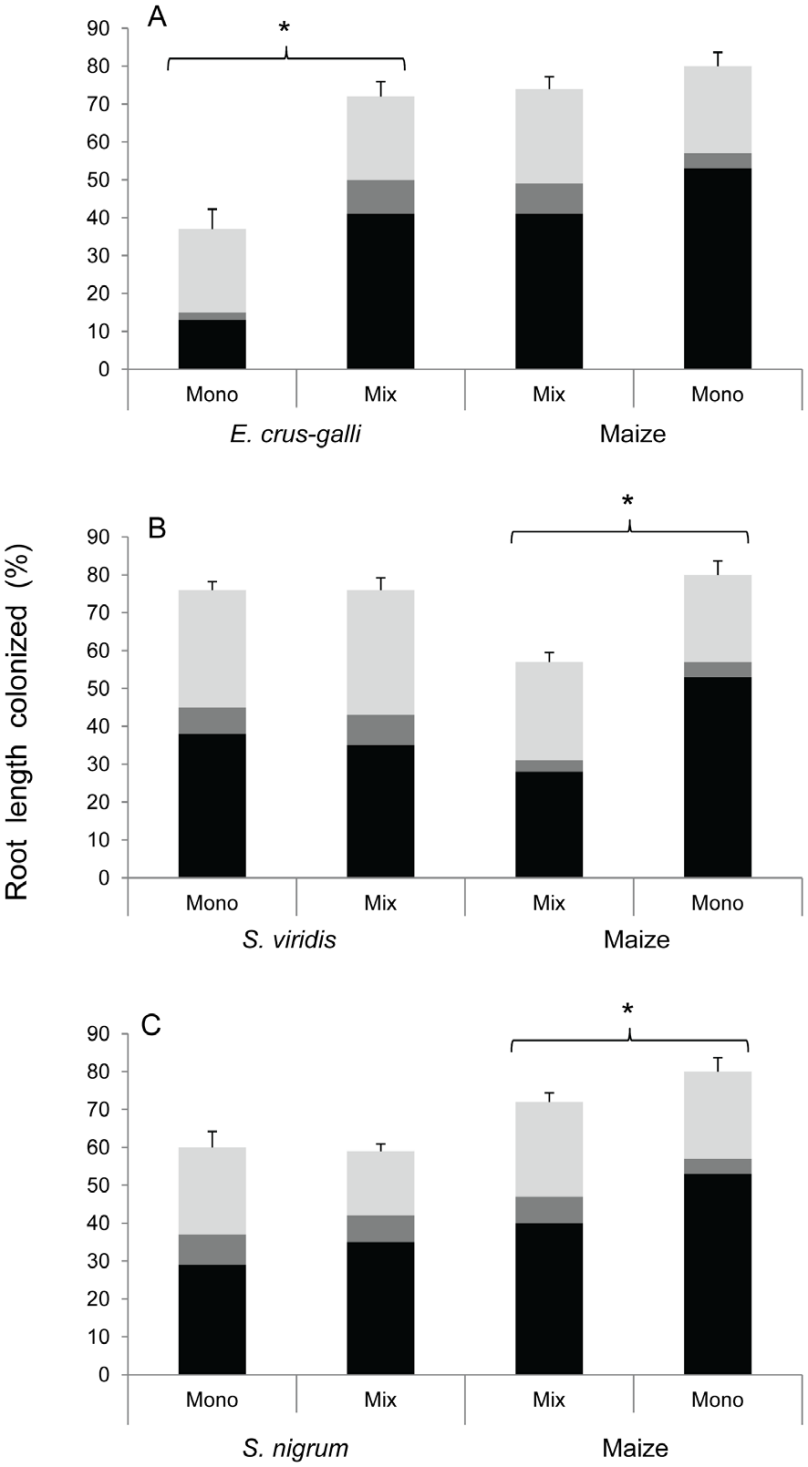
Percentage of total root length colonized by AMF in experiment 2. Total root length colonized by AMF (%) is represented as the sum of the percentages of root length colonized by arbuscules (black), vesicles (dark grey) and hyphae (light grey). Plant species were grown alone (Mono) or in weed-maize combinations (Mix). Bars are means of seven replicates ± SEM. Asterisks represent significant differences in total root colonization between monocultures and mixtures, for each species. Root colonization of maize in monoculture repeated in each graph for better visual interpretation.

The presence of maize had a differential effect on the percentage of root length colonized by AMF for the three weed species (significant “plant combination”×“weed species” interaction *F*_2,36_ = 12.4, *P* = 0.0001). The weed species *S. viridis* and *S. nigrum* achieved comparably high levels of colonization in the absence and in the presence of maize (Fig. 2B, C). On the contrary, *E. crus-galli* showed a significant increase of total root length colonized by AMF, from 37±5.3% in monoculture to 72±3.9% when grown in mixture with maize (*t* = 5.4, *P*<0.0001; Fig. 2A).

In general, maize achieved a significantly higher AMF root colonization when grown alone (80±3.7%) than when coexisting with weeds (68±2.3%; *F*_1,26_ = 9.1, *P*<0.01; effect measured across the three weed species). AMF root colonization of maize was consistently reduced in coexistence with each of the weed species (Fig. 2) but not significantly in the presence of *E. crus-galli* (*t* = −1.6, *P* = 0.134; Fig. 2A).

#### Growth responses

The presence of maize had also a differential effect on the mycorrhizal growth responses of the three weed species (significant “plant combination”×“weed species” interaction *F*_2,36_ = 15.4, *P*<0.0001).

**Figure 3:**
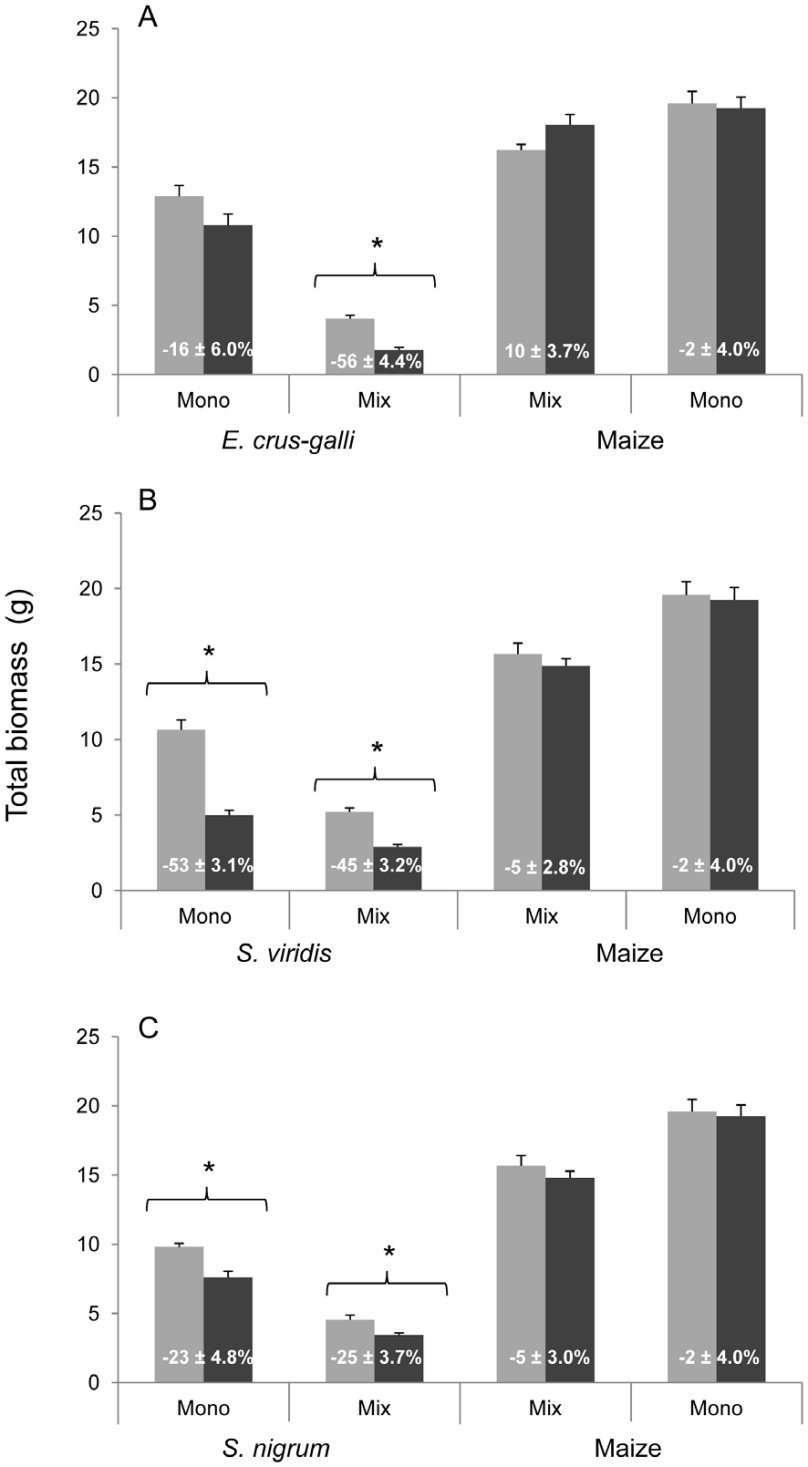
Total biomass (g) of the three weed species and maize in experiment 2. Plant species were grown alone (Mono) or in weed-maize combinations (Mix). Monocultures and mixtures of *E. crus-galli* and maize (**A**), *S. viridis* and maize (**B**) and *S. nigrum* and maize (**C**) were inoculated with AMF (black bars) or with NM control inoculum (grey bars). Bars are means of seven replicates ± SEM. Asterisks represent significant differences in total biomass between the AMF treatment and NM control for each species, in monocultures or mixtures. Total biomass of maize in monoculture repeated in each graph for better visual interpretation. Values in white inserted in the bars indicate the mycorrhizal growth response (%) of each species, in monocultures or mixtures (mean of seven replicates ± SEM).

Consistent with the previous experiment, in monocultures, the three weed species all responded negatively to AMF (Fig. 3). These mycorrhizal growth responses represented a significant reduction of total biomass in the presence of AMF, compared to NM controls, in the case of *S. viridis* (*t* = −7.7, *P*<0.0001; Fig. 3B) and *S. nigrum* (*t* = −4.1, *P*<0.001; Fig. 3C) but not in *E. crus-galli* (*t* = −1.8, *P* = 0.075, Fig. 3A).

In the presence of maize, the three weed species also responded negatively to AMF (Fig. 3). The total biomass of each weed was significantly reduced in the AMF treatment as compared to the NM controls (*t* = −7.1, *P*<0.0001 for *S. viridis*; *t* = −3.4, *P*<0.01 for *S. nigrum* and *t* = −6.9, *P*<0.0001 for *E. crus-galli*). However, the weed *E. crus-galli* showed a significantly amplified negative mycorrhizal growth response when coexisting with maize (*t* = −5.3, *P*<0.0001; Fig. 3A), compared to the respective monoculture, while *S. viridis* and *S. nigrum* responded similarly (*P*>0.05), with or without maize (Fig. 3B, C).

Maize was insensitive to the presence of AMF when grown alone (*F*_1,12_ = 0.08, *P* = 0.783) (Fig. 3), showing a mycorrhizal growth response of −2±4.0%. Similarly, when weeds were present, there was no significant difference in maize biomass between the AMF treatment and the NM controls (*F*_1,40_ = 0.01, *P* = 0.923; effect measured across the three weed species), as reflected by the neutral (0.3±2.3%) mycorrhizal growth response of maize in mixtures. Nonetheless, the mycorrhizal growth response of maize grown with *E. crus-galli* was significantly different from that of the monoculture, increasing to 10±3.7% (*t* = 2.3, *P*<0.05), and represented a marginally significant increase of maize total biomass in the presence of AMF, compared to the NM controls (*t* = 2.1, *P* = 0.057; Fig. 3A).

## Discussion

Our results show that (1) biomass of four of the investigated weed species was significantly reduced by *G. intraradices* while none of the weeds significantly benefited from inoculation with this AMF, (2) growth of the weed species *E. crus-galli*, *S. viridis* and *S. nigrum* was also reduced by a combination of AMF and (3) the presence of a crop (maize) further amplified the negative effect of AMF in one out of the three weed species tested.

Previous work had shown that some weeds responded negatively to soil fungi [28], [29] and that weeds grown together with a crop can be suppressed by AMF [30]. In this study we show that monocultures of some troublesome weeds in Switzerland and Europe respond negatively to inoculation with the AMF species *G. intraradices*. Moreover, the weed species *E. crus-galli*, *S. viridis* and *S. nigrum* maintained negative mycorrhizal growth responses when inoculated with a mixture of *G. intraradices*, *G. mosseae* and *G. claroideum*. Earlier experiments have shown that ruderal plant species respond negatively to AMF [23], [24]. Most weeds originate from ruderal habitats [50] and our results thus confirm that many plants from this type of habitat do not establish a beneficial AM symbiosis. However, although this may be generally true for ruderal plants as a group, not all weed species responded in this way in our study. For instance, two of the weeds tested in experiment 1, *P. annua* and *A. spica-venti*, showed positive, albeit not statistically significant, mycorrhizal growth responses above 20%. Hence, it is not possible to generalize our results to all weeds. In addition, plant responses to AMF depend on the identity of the fungus [12], [37], indicating that the composition and diversity of AMF communities are likely to affect AMF-plant interactions. Future experiments should test the effect of whole AMF communities (with differential composition and diversity) on weed growth and whether specific weed species are absent or scarcer in fields with high abundance of AMF and/or with a specific composition of AMF communities.

Clover significantly benefited in biomass production from inoculation with the AMF species *G. intraradices*. On the other hand, the two cereal crops showed a negative mycorrhizal growth response that, in the case of maize, represented a significant growth reduction in the presence of *G. intraradices*. This is not unexpected as wheat and maize are often unresponsive or negatively responsive to AMF colonization in pot experiments where root development is space limited [18], [51]–[53], while clover is mostly positively responsive [11], [40]. When inoculated with a combination of AMF species, the biomass of maize in monoculture was not anymore significantly reduced most probably due to differences in AMF diversity and abiotic conditions between the experiments. In the presence of weeds, maize was also overall unresponsive while the coexisting weed species, in contrast, grew significantly less when colonized by AMF. This suggests that maize forms a more beneficial symbiosis with AMF than the tested weed species as it has been observed for another crop, sunflower [30]. Moreover, the competitive interactions of the different plants for soil nutrients, mediated by the AMF networks, might have resulted in further deprivation of symbiotic benefits to the weeds as compared to the maize plants. This latter hypothesis will need to be further tested using isotopic tracers in the future.

The suppressive effect of a combination of AMF species on *S. viridis* and *S. nigrum* was comparable between monocultures and mixtures with maize. In contrast, the negative mycorrhizal growth response of *E. crus-galli* was significantly amplified (by 40%), from −16% in monoculture to −56% when in coexistence with maize. Interestingly, also only in *E. crus-galli* the percentage of root length colonized by AMF differed between monocultures and mixtures, increasing from 37% to 72% when maize was present. This indicates dynamic shifts of symbiotic costs and benefits in a plant community sharing a common mycorrhizal network. This phenomenon may well be of interest to practical exploitation in agriculture, but its mechanistic understanding remains rather poor. The fact that the amplified negative mycorrhizal growth response in *E. crus-galli* in the presence of maize was only observed when accompanied by an increase in AMF root colonization suggests that enhanced colonization levels might have been responsible for the latter. Previously, only species with a percentage of root length colonized by *G. intraradices* higher than 50% showed a negative mycorrhizal growth response (Fig. 1). On the other hand, unlike in the work of Li et al. [53] and Grace et al. [20], none of the plant species poorly colonized by AMF showed significant growth depressions when grown in monocultures. Therefore, our results suggest that high AMF root colonization might be an important factor determining negative mycorrhizal growth responses, at least under our experimental conditions. It is important to note that in other studies performed with different plant species and in different conditions, highly colonized plants could still strongly benefit from AMF [38], [41], [54]. We are currently investigating whether the negative effect of AMF on some weeds depends on AMF abundance in their roots.

Often, mycorrhizal growth depressions are attributed to AMF parasitism, where carbon (C) demand from the fungus exceeds the benefits of increased nutrient uptake. In agreement with this notion, Graham & Abbott [18] found lower sucrose concentrations in the roots of negatively responsive wheat plants colonized by aggressive AMF. Our own results seem to fit in this explanation as growth depressions were associated with high root colonization and hence potentially high fungal C costs. Moreover, the soil substrate used was P-rich, and high nutrient availability generally increases the likelihood of parasitic associations [10]. In other situations though, when growth depressions occur in weakly colonized plants [20], [53], C drain might not be a satisfactory explanation and alternative mechanisms have been suggested. These include: reduction or suppression of the direct plant P uptake pathway with no or insufficient compensation from the AMF uptake pathway [53], allelopathic effects of fungal exudates [23] and AMF induction of costly plant defense responses [55]. In addition, AMF-mediated plant competition can cause growth depressions in positively responsive plants in the absence of a competitor [39]. However, it cannot alone explain the negative mycorrhizal growth responses of the weeds grown with maize as their responses were also negative in the absence of the crop.

It is difficult to assess the impact of AMF on plants in the field because AMF are usually already present in the soil and their abundance cannot be easily manipulated without simultaneously changing other factors or activity of other organisms [56]. For this reason, our experiments were established under controlled conditions in sterilized soil to which AMF were added (together with the associated microbial communities). In this way, it was possible to successfully manipulate the presence of AMF, in line with previous experiments [57]–[60]. Although a recent study by Pringle & Bever [61] has shown comparable effects of AMF on plants grown in growth chambers and in an open field, it is important to consider that our study design with sterilized soil, absence of larger soil organisms such as nematodes and earthworms, for instance, and manipulation of microcosms under glasshouse conditions has limitations and theoretically does not reflect conditions found in the field [56]. In agreement, we observed that some of the weed species investigated in this study seemed to be generally less colonized by AMF in the field (Table S9) than in our glasshouse experiments. Therefore, despite some experimental constraints, future work investigating AMF-weed interactions should be performed in field conditions in order to enhance agricultural/ecological realism.

The results obtained here certainly do not indicate a strong potential for AMF as a weed biocontrol agent. Even if AMF suppressed some of the investigated weed species, the weeds kept producing some biomass and several species started to produce flowers or set seeds. Moreover, as previously mentioned, not all the weed species tested were negatively affected by AMF. The differential weed responses to AMF indicate though that the composition of weed communities in agricultural fields and the relative abundances of positively and negatively responsive species within these communities, can be altered by AMF, similar to what has been observed in other studies with different plant species [37], [62]. Furthermore, our results are also relevant for ruderal plant communities since several of the investigated weeds are abundant in heavily disturbed sites.

The suppressive effect of AMF on the growth of some weeds, especially in coexistence with crop plants, might be of particular interest to more sustainable farming systems, where weed management to tolerable levels rather than total weed eradication is the prevailing strategy. Previous studies have shown that AMF provide a number of beneficial ecosystem services [63], [64]. If our results can be confirmed in field conditions, they provide an additional argument to promote AMF activity in agroecosystems.

## Supporting Information

Table S1 pH and primary plant-available nutrient concentrations of the autoclaved soil substrate used in each experiment.
(DOC)

Table S2 Total biomass (g) of each plant species inoculated with *G. intraradices* (AMF) or with NM control inoculum, in experiment 1.
(DOC)

Table S3 Results of the ANOVA testing for the effects of diverse plant species on the total root length colonized (RLC) by AMF and on the mycorrhizal growth response (MGR) in experiment 1.
(DOC)

Table S4 Results of the ANOVA testing for the effects of AMF and plant species on the total biomass in experiment 1.
(DOC)

Table S5 Results of the ANOVA testing for the effects of plant combination and species on the total root length colonized (RLC) by AMF and on the mycorrhizal growth response (MGR) of weeds in experiment 2.
(DOC)

Table S6 Results of the ANOVA testing for the effects of AMF and species on the total biomass of weeds grown in monocultures or mixtures with maize in experiment 2.
(DOC)

Table S7 Results of the ANOVA testing for the effects of plant combination on the total root length colonized (RLC) by AMF and on the mycorrhizal growth response (MGR) of maize in experiment 2.
(DOC)

Table S8 Results of the ANOVA testing for the effects of AMF on the total biomass of maize grown in monoculture or mixtures with weeds in experiment 2.
(DOC)

Table S9 Percentage of root length colonized by AMF in the field.
(DOC)
