# Supplementary material for: Can Arbuscular Mycorrhizal Fungi Reduce the Growth of Agricultural Weeds?
Source: PLoS One. 2011 Dec 2;6(12):e27825. doi: 10.1371/journal.pone.0027825 (PMC3229497; doi:10.1371/journal.pone.0027825)
Supplement: Table S1 — pH and primary plant-available nutrient concentrations of the autoclaved soil substrate used in each experiment. (DOC) [file pone.0027825.s001.doc]

**Table S1.** pH and primary plant-available nutrient concentrations of the autoclaved soil substrate used in each experiment.

|  | Exp 1 | Exp 2 |
| --- | --- | --- |
| pH (H_2_O) | 7.7 | 8.1 |
| Water soluble NO_3_^-^ and NH_4_^+^ | 53.3 mg kg^–1^ | 38.3 mg kg^–1^ |
| P_2_O_5_ (CO_2_-saturated water extracted) | 21 mg kg^–1^ | 20 mg kg^–1^ |
| K_2_O (CO_2_-saturated water extracted) | 56 mg kg^–1^ | 140 mg kg^–1^ |

Water-soluble inorganic N (NO_3_^-^ and NH_4_^+^) was determined with a Skalar segment flow analyzer.
