# Supplementary material for: Can Arbuscular Mycorrhizal Fungi Reduce the Growth of Agricultural Weeds?
Source: PLoS One. 2011 Dec 2;6(12):e27825. doi: 10.1371/journal.pone.0027825 (PMC3229497; doi:10.1371/journal.pone.0027825)
Supplement: Table S2 — Total biomass (g) of each plant species inoculated with G. intraradices (AMF) or with NM control inoculum, in experiment 1. (DOC) [file pone.0027825.s002.doc]

**Table S2.** Total biomass (g) of each plant species inoculated with *G. intraradices* (AMF) or with NM control inoculum, in experiment 1.

|  | Total biomass (g) | |  |  |
| --- | --- | --- | --- | --- |
| **Crop species** | NM | AMF | *t*-value | *P*-value |
| Clover | 1.1 ± 0.1 | 1.8 ± 0.1 | 2.5 | **0.016** |
| Maize | 5.2 ± 0.3 | 4.1 ± 0.2 | -3.7 | **< 0.001** |
| Wheat | 1.6 ± 0.2 | 1.3 ± 0.1 | -1.0 | 0.324 |
| **Weed species** |  |  |  |  |
| *Agropyron repens* | 2.0 ± 0.2 | 1.8 ±0.2 | -0.4 | 0.658 |
| *Alopecurus myosuroides* | 1.8 ± 0.3 | 1.8 ± 0.2 | 0.3 | 0.771 |
| *Apera spica-venti* | 0.6 ± 0.7 | 0.9 ± 0.1 | 1.0 | 0.318 |
| *Cirsium arvense* | 2.2 ± 0.1 | 1.7 ± 0.4 | -1.7 | 0.093 |
| *Digitaria sanguinalis* | 4.6 ± 0.2 | 3.6 ± 0.2 | -3.5 | **< 0.001** |
| *Echinochloa crus-gali* | 4.7 ± 0.4 | 3.1 ± 0.3 | -5.7 | **< 0.0001** |
| *Poa annua* | 0.4 ± 0.04 | 0.6 ± 0.1 | 0.7 | 0.486 |
| *Setaria viridis* | 4.6 ± 0.2 | 3.4 ± 0.3 | -4.3 | **< 0.0001** |
| *Solanum nigrum* | 2.6 ± 0.1 | 1.9 ± 0.1 | -2.5 | **0.014** |

Values are means of six replicates ± SEM except for *A. repens* inoculated with NM control inoculum where the presented value is a mean of five replicates ± SEM. *P*-values in bold represent significant differences in total biomass between AMF treatment and NM control for each plant species.
