# Supplementary material for: Can Arbuscular Mycorrhizal Fungi Reduce the Growth of Agricultural Weeds?
Source: PLoS One. 2011 Dec 2;6(12):e27825. doi: 10.1371/journal.pone.0027825 (PMC3229497; doi:10.1371/journal.pone.0027825)
Supplement: Table S3 — Results of the ANOVA testing for the effects of diverse plant species on the total root length colonized (RLC) by AMF and on the mycorrhizal growth response (MGR) in experiment 1. (DOC) [file pone.0027825.s003.doc]

**Table S3.** Results of the ANOVA testing for the effects of diverse plant species on the total root length colonized (RLC) by AMF and on the mycorrhizal growth response (MGR) in experiment 1.

|  | RLC (total) | | |  | MGR | | |
| --- | --- | --- | --- | --- | --- | --- | --- |
| Source of variation | df | *F* | *P* |  | df | *F* | *P* |
| Plant species | 11 | 298.7 | < 0.0001 |  | 11 | 9.0 | < 0.0001 |
| Error | 60 |  |  |  | 60 |  |  |
