# Supplementary material for: Can Arbuscular Mycorrhizal Fungi Reduce the Growth of Agricultural Weeds?
Source: PLoS One. 2011 Dec 2;6(12):e27825. doi: 10.1371/journal.pone.0027825 (PMC3229497; doi:10.1371/journal.pone.0027825)
Supplement: Table S4 — Results of the ANOVA testing for the effects of AMF and plant species on the total biomass in experiment 1. (DOC) [file pone.0027825.s004.doc]

**Table S4.** Results of the ANOVA testing for the effects of AMF and plant species on the total biomass in experiment 1.

|  | Total biomass | | |
| --- | --- | --- | --- |
| Source of variation | df | *F* | *P* |
| AMF | 1 | 29.3 | < 0.0001 |
| Plant species | 11 | 93.2 | < 0.0001 |
| AMF × Plant species | 11 | 6.1 | < 0.0001 |
| Error | 119 |  |  |
