# Supplementary material for: Can Arbuscular Mycorrhizal Fungi Reduce the Growth of Agricultural Weeds?
Source: PLoS One. 2011 Dec 2;6(12):e27825. doi: 10.1371/journal.pone.0027825 (PMC3229497; doi:10.1371/journal.pone.0027825)
Supplement: Table S5 — Results of the ANOVA testing for the effects of plant combination and species on the total root length colonized (RLC) by AMF and on the mycorrhizal growth response (MGR) of weeds in experiment 2. (DOC) [file pone.0027825.s005.doc]

**Table S5.** Results of the ANOVA testing for the effects of plant combination and species on the total root length colonized (RLC) by AMF and on the mycorrhizal growth response (MGR) of weeds in experiment 2.

|  | RLC (total) | | |  | MGR | | |
| --- | --- | --- | --- | --- | --- | --- | --- |
| Source of variation | df | *F* | *P* |  | df | *F* | *P* |
| Plant combination | 1 | 4.7 | 0.038 |  | 1 | 1.1 | 0.303 |
| Weed species | 2 | 23.7 | < 0.0001 |  | 2 | 23.1 | < 0.0001 |
| Plant combination × Weed species | 2 | 12.4 | 0.0001 |  | 2 | 15.4 | < 0.0001 |
| Error | 36 |  |  |  | 36 |  |  |
