# Supplementary material for: Can Arbuscular Mycorrhizal Fungi Reduce the Growth of Agricultural Weeds?
Source: PLoS One. 2011 Dec 2;6(12):e27825. doi: 10.1371/journal.pone.0027825 (PMC3229497; doi:10.1371/journal.pone.0027825)
Supplement: Table S6 — Results of the ANOVA testing for the effects of AMF and species on the total biomass of weeds grown in monocultures or mixtures with maize in experiment 2. (DOC) [file pone.0027825.s006.doc]

**Table S6.** Results of the ANOVA testing for the effects of AMF and species on the total biomass of weeds grown in monocultures or mixtures with maize in experiment 2.

|  | Total biomass monocultures | | |  | Total biomass mixtures | | |
| --- | --- | --- | --- | --- | --- | --- | --- |
| Source of variation | df | *F* | *P* |  | df | *F* | *P* |
| AMF | 1 | 63.7 | < 0.0001 |  | 1 | 102.0 | < 0.0001 |
| Weed species | 2 | 18.2 | < 0.0001 |  | 2 | 15.1 | < 0.0001 |
| AMF × Weed species | 2 | 7.6 | 0.0017 |  | 2 | 4.3 | 0.021 |
| Error | 36 |  |  |  | 36 |  |  |
