# Supplementary material for: Can Arbuscular Mycorrhizal Fungi Reduce the Growth of Agricultural Weeds?
Source: PLoS One. 2011 Dec 2;6(12):e27825. doi: 10.1371/journal.pone.0027825 (PMC3229497; doi:10.1371/journal.pone.0027825)
Supplement: Table S7 — Results of the ANOVA testing for the effects of plant combination on the total root length colonized (RLC) by AMF and on the mycorrhizal growth response (MGR) of maize in experiment 2. (DOC) [file pone.0027825.s007.doc]

**Table S7.** Results of the ANOVA testing for the effects of plant combination on the total root length colonized (RLC) by AMF and on the mycorrhizal growth response (MGR) of maize in experiment 2.

|  | RLC (total) | | |  | MGR | | |
| --- | --- | --- | --- | --- | --- | --- | --- |
| Source of variation | df | *F* | *P* |  | df | *F* | *P* |
| Plant combination ^1^ | 1 | 9.1 | 0.006 |  | 1 | 0.1 | 0.737 |
| Error ^1^ | 26 |  |  |  | 26 |  |  |
| Plant combination ^2^ | 3 | 11.2 | < 0.0001 |  | 3 | 3.5 | 0.03 |
| Error ^2^ | 24 |  |  |  | 24 |  |  |

^1^ Plant combination treated as a factor with two levels: maize monoculture and mixture with weeds

^2^ Plant combination treated as a factor with four levels: maize monoculture, maize in mixture with *E. crus-galli*, maize in mixture with *S. viridis* and maize in mixture with *S. nigrum*
