# Supplementary material for: Can Arbuscular Mycorrhizal Fungi Reduce the Growth of Agricultural Weeds?
Source: PLoS One. 2011 Dec 2;6(12):e27825. doi: 10.1371/journal.pone.0027825 (PMC3229497; doi:10.1371/journal.pone.0027825)
Supplement: Table S8 — Results of the ANOVA testing for the effects of AMF on the total biomass of maize grown in monoculture or mixtures with weeds in experiment 2. (DOC) [file pone.0027825.s008.doc]

**Table S8.** Results of the ANOVA testing for the effects of AMF on the total biomass of maize grown in monoculture or mixtures with weeds in experiment 2.

|  | Total biomass monoculture | | |  | Total biomass mixtures | | |
| --- | --- | --- | --- | --- | --- | --- | --- |
| Source of variation | df | *F* | *P* |  | df | *F* | *P* |
| AMF | 1 | 0.08 | 0.783 |  | 1 | 0.01 | 0.923 |
| Error | 12 |  |  |  | 40 |  |  |
