# Supplementary material for: Can Arbuscular Mycorrhizal Fungi Reduce the Growth of Agricultural Weeds?
Source: PLoS One. 2011 Dec 2;6(12):e27825. doi: 10.1371/journal.pone.0027825 (PMC3229497; doi:10.1371/journal.pone.0027825)
Supplement: Table S9 — Percentage of root length colonized by AMF in the field. (DOC) [file pone.0027825.s009.doc]

**Table S9.** Percentage of root length colonized by AMF in the field.

|  |  | Root length colonized (%) | | |
| --- | --- | --- | --- | --- |
| Weed species | Field | Total | Vesicles | Arbuscules |
| *Alopecurus myosuroides* | a | 19 | 4 | 7 |
|  | a | 24 | 7 | 4 |
|  | b* | 15 | 4 | 2 |
|  | b* | 24 | 2 | 11 |
|  | b* | 36 | 6 | 8 |
| *Echinochloa crus-galli* | c | 25 | 1 | 12 |
|  | c | 23 | 3 | 11 |
|  | c | 14 | 1 | 8 |
|  | a | 12 | 1 | 2 |
|  | a | 10 | 4 | 3 |
|  | a | 10 | 3 | 3 |
|  | d* | 7 | 1 | 2 |
|  | d* | 12 | 1 | 3 |
|  | d* | 25 | 2 | 11 |
|  | e | 6 | 0 | 3 |
|  | e | 9 | 1 | 2 |
|  | e | 15 | 4 | 4 |
| *Poa annua* | c | 13 | 3 | 3 |
|  | c | 18 | 4 | 3 |
|  | c | 25 | 4 | 8 |
| *Solanum nigrum* | f | 12 | 2 | 3 |
|  | f | 15 | 5 | 4 |
|  | f | 14 | 3 | 1 |
|  | g | 17 | 4 | 4 |

Weeds were collected in July 2011 in five organic and two conventional (fields b and d, marked with an asterisk) maize fields in Switzerland (field a: 47°25'50.18'' N, 8°44''31.12'' E; field b: 47°37'44.00'' N, 8°45''56.36'' E; field c: 47°31'06.90'' N, 8°18''29.18'' E; field d: 47°32'48.45'' N, 8°40''08.83'' E; field e: 47°24'54.61'' N, 8°12''42.43'' E; field f: 47°32'34.34'' N, 8°59''40.12'' E; field g: 47°37'56.44'' N, 8°45''26.30'' E). All fields were ploughed in Spring 2011 before maize was sown. All fields were organically fertilized and fields b and d received additionally one application of pure synthetic urea. Fertilization ranged from 74 (field a) to 160 (field d) kg ha^-1^ water soluble N, 63 (field b) to 234 (field e) kg ha^-1^ P_2_O_5_ and 133 (field b) to 445 (field a) kg ha^-1^ K_2_O. When possible three individual plants from a species were collected in the fields where that species was present. Roots were separated from shoots, thoroughly washed, cut into 1 cm segments, mixed and a subsample taken to determine the percentage of root length colonized by AMF (as described in Material and Methods). Percentage of total root length colonized by AMF and respective partial percentages of vesicles and arbuscules are given per individual plant.
